# Does texting while walking really affect gait in young adults?

**DOI:** 10.1186/s12984-015-0079-4

**Published:** 2015-09-22

**Authors:** Valentina Agostini, Francesco Lo Fermo, Giuseppe Massazza, Marco Knaflitz

**Affiliations:** Dipartimento di Elettronica e Telecomunicazioni, Politecnico di Torino, Corso Duca degli Abruzzi 24, 10129 Torino, Italy; Università degli Studi di Torino, Via Zuretti 29, 10126 Torino, Italy; SC Medicina Fisica e Riabilitazione U, AO Città della Salute e della Scienza, Corso Bramante 82, 10126 Torino, Italy

**Keywords:** Gait, Gait analysis, Texting and walking, Muscle co-contraction, EMG, Smartphone use, Dual task

## Abstract

**Background:**

Texting on a smartphone while walking has become a customary task among young adults. In recent literature many safety concerns on distracted walking have been raised. It is often hypothesized that the allocation of attentional resources toward a secondary task can influence dynamic stability. In the double task of walking and texting it was found that gait speed is reduced, but there is scarce evidence of a modified motor control strategy compromising stability. The aim of this study is twofold: 1) to comprehensively examine the gait modifications occurring when texting while walking, including the study of the lower limb muscle activation patterns, 2) to specifically assess the co-contraction of ankle antagonist muscles. We hypothesized that texting while walking increases co-contractions of ankle antagonist muscles when the body weight is transferred from one lower limb to the other, to improve the distal motor control and joint stabilization.

**Methods:**

From the gait data collected during an instrumented walk lasting 3 min, we calculated the spatio-temporal parameters, the ankle and knee kinematics, the muscle activation patterns of tibialis anterior, gastrocnemius lateralis, peroneus longus, rectus femoris, and lateral hamstrings, and the co-contraction (occurrence and duration) of the ankle antagonist muscles (tibialis anterior and gastrocnemius lateralis), bilaterally.

**Results:**

Young adults showed, overall, small gait modifications that could be mainly ascribable to gait speed reduction and a modified body posture due to phone handling. We found no significant alterations of ankle and knee kinematics and a slightly delayed activation onset of the left gastrocnemius lateralis. However, we found an increased co-contraction of tibialis anterior and gastrocnemius lateralis, especially during mid-stance. Conversely, we found a reduced co-contraction during terminal stance.

**Conclusions:**

Our results suggest that, in young adults, there is an adjustment of the motor control strategy aimed at increasing ankle joint stability in a specific and “critical” phase of the gait cycle, when the body weight is transferred from one leg to the other.

## Background

Young individuals rarely just walk. They are frequently engaged in additional tasks, such as talking on a mobile phone, listening to music or texting messages. Emerging research evidenced the dangers of distracted walking and reduced situation awareness in pedestrians using smartphones [1]. In particular, it was reported that texting on a smartphone creates a significantly greater interference effect on walking than talking or reading [2, 3]. As a matter of fact, the activity of texting while walking is a more complex task, since it usually integrates visual-motor coordination, bimanual movements for tapping with thumbs of both hands, and cognitive attention to the message content. A recent study showed that, for what concerns their frontal plane margin of stability, experienced texters are more affected by the physical than by the cognitive demand of texting [4]. Subjects may try to control foot placement and joint kinematics during cell phone use or another cognitive task with a visual component, to ensure sufficient dynamic margins of stability [5].

Existing research provided insight into spatio-temporal parameter modifications of texting while walking and, usually, a reduced gait speed was reported [2, 3, 6, 7]. Furthermore, stride-to-stride variability was found to be increased in several dual task experiments involving cognitive-demanding tasks [8–10]. However, writing on a smartphone while walking involves both cognitive and physical resources, the integration of gross and fine motor functions, near and far vision. Hence, stride-to-stride variability might be even further increased.

Previous research provided evidence that individuals, while texting, have altered head and trunk kinematics [3], since their head is almost inevitably inclined forwards to read the display. However, little is known on how the Central Nervous System (CNS) adapts to control lower limbs and increase stability, and to what extent, and how, young adults modify their motor scheme during the dual task of texting and walking. More specifically, none of the existing studies reported gait adaptations in terms of ankle and knee joint kinematics, lower limb muscle activation patterns, and co-contraction of ankle antagonist muscles.

Recent literature on the detection of muscle activation timing from the surface electromyographic (EMG) signal highlighted the importance of using innovative methods, known under the name of “statistical gait analysis”, to properly handle the large intra- and inter-subject variability of human gait [11–15]. These methods may constitute a valuable analysis tool when small changes in the muscle activation patterns are expected [13, 15], as it may happen in dual-task protocols evaluating the walking function with and without some additional task. However, to the best of our knowledge, they have never been applied within this context.

Muscle co-contraction is the simultaneous activation of agonist and antagonist muscles crossing a joint [16] and its function is to increase joint stiffness. A recent study on young adults showed that tibialis anterior (TA) and gastrocnemius lateralis (GL) act as pure agonist/antagonists for ankle plantar/dorsiflexion (no co-contraction) in only 21 % of strides [17]. In the remaining strides, co-contractions appeared, both in stance and/or swing, with the probable function of improving balance and control ankle stability. It is known that attentional resources toward a secondary cognitive task can lead to a diminished ankle proprioceptive performance [18]. Hence, we hypothesized that texting while walking increases co-contractions of ankle antagonist muscles when the body weight is transferred from one lower limb to the other, to improve the distal motor control and joint stabilization.

The purpose of this study was to comprehensively examine, in a population of young adults, the gait modifications due to texting on a smartphone while walking, with a focus on distal motor control. Along with spatio-temporal parameters and stride-to-stride variability, we analyzed, bilaterally: 1) ankle and knee kinematics, 2) the muscle activation patterns of five lower limb muscles, 3) the co-contraction of TA/GL muscles.

## Methods

### Participants

Eighteen healthy young adults, aged from 20 to 30 years, with normal or corrected-to-normal vision, were recruited from the university community (8 males/10 females, height: 1.69 ± 0.08 m; weight: 63.3 ± 10 kg). Participants were eligible if they used, on a daily basis, a smartphone with a display between 3.5 and 5 inches, with a touch screen and virtual QWERTY keyboard, and had more than 2 months experience with their current phone. Individuals reporting neurological, musculoskeletal disorders or other conditions that could affect their gait or capacity of typing were excluded from the study.

This study was approved by the local Institutional Review Board and all procedures conformed to the Helsinki declaration. Written informed consent was obtained by all participants.

### Procedures

**Fig. 1:**
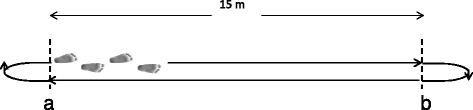
Walking path. Subjects are instructed to pass the marks (**a**, **b**) before decelerating and turning back

Participants were assessed in a well-lit room, over a straight path of 15 m. Subjects were asked to walk back and forth along the path, at their natural pace, for 3 min (Fig. 1). We examined 2 different conditions: a) walking, b) walking and texting. The two conditions were administered randomly. In condition b) no instruction was given on task prioritization to better reproduce a real-world situation. Participants used their own smartphone and their usual typing method (one or two hands). They were asked to type a message describing their own activities on the day before the test. After the test completion, they were asked to send the message to the experimenter, so that he could count the total number of characters written during the 3 min, in order to estimate the average typing speed, calculated as the number of characters per minute.

The experimenter timed each subject’s passage through the 15-m walkway (see Fig. 1), with the exclusion of direction changes. More specifically, he measured the time that the subject walked from A to B, then from B to A, then from A to B again, etc.…. Gait speed stability among the different A-B passages was checked and the average gait speed was defined as the total distance walked in a straight line divided by the total time required to go through it.

Subjects walked barefooted, with thin foot-switches placed under the foot-soles (size: 10 mm × 10 mm × 0.5 mm; activation force: 3 N), beneath the first and fifth metatarsal heads, and beneath the back portion of the heel. Sagittal plane electro-goniometers were placed at ankle and knee joints (accuracy: 0.5 deg). Surface EMG probes were placed over tibialis anterior (TA), gastrocnemius lateralis (GL), peroneus longus (PL), rectus femoris (RF), and lateral hamstring (LH), bilaterally. EMG probes were active and utilized Ag-disks (diameter: 4-mm, inter-electrode distance: 12 mm). The signal amplifier had a gain of 1000 and a 3-dB bandwidth from 10 Hz to 400 Hz. The sampling frequency was 2 kHz and the signals were converted by a 12-bit analog to digital converter. Signals detected by sensors on the subject and a synchronized digital video were recorded by the system STEP32, Medical Technology - DemItalia (Italy).

Since in correspondence of the turns participants had to decelerate, change directions, and reinitiate a forward directed trajectory that involved an acceleration phase, the strides corresponding to direction changes were automatically removed by the system software.

### Data analysis

**Fig. 2:**
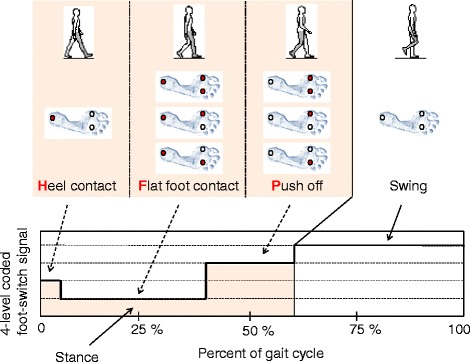
Gait phases. Foot-switch signal coding (right foot). A red circle under the foot sole indicates a closed foot-switch. The signal has 4 quantization levels: 1) only the heel foot-switch is closed (Heel contact), 2) the heel foot-switch is closed, and at least one of the foot-switches under the forefoot is also closed (Flat foot contact), 3) the heel foot-switch is open, and at least one of the foot-switches under the forefoot is closed (Push off), 4) all the foot-switches are open (Swing)

In each test condition, for each patient, an average of 157 ± 11 gait cycles were analyzed. For each lower limb, time events were identified using a 4-level footswitch signal, coded as follows: 1) heel footswitch closed, 2) heel- and (at least one) forefoot-switch also closed, 3) at least one forefoot switch closed, 4) no footswitches closed [19]. The following gait phases were determined: heel contact (H), flat-foot contact (F), push-off/heel-off (P) and swing (Fig. 2). We calculated the duration of the sub-phases of stance H, F, P expressed as percentage of the gait cycle (% GC).

The stride-to-stride variability was assessed by the coefficient of variation (CV) of the stride time, defined as follows:

1 $$ CV\; of\; stride\kern0.24em  time\;\left(\%\right)=\frac{standard\; deviation\;\left( stride\kern0.24em  time\right)}{mean\;\left( stride\kern0.24em  time\right)}\cdot 100 $$

Dual task effect (DTE) on gait parameters was calculated as the relative change in performance in the dual-task condition compared to single-task performance:

2 $$ DTE=\frac{\left| single\kern0.24em  task-\left. dual\kern0.24em  task\right|\right.}{single\kern0.24em  task}\cdot 100 $$

**Fig. 3:**
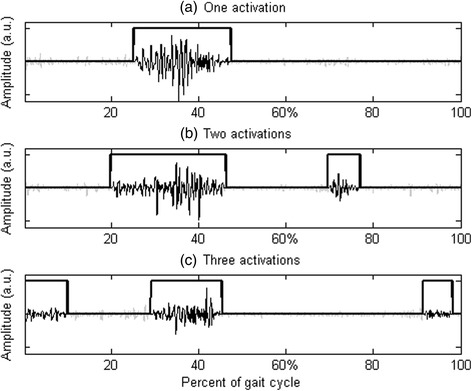
EMG signal: detection of muscle activation intervals. Examples of gastrocnemius lateralis activation patterns in three different strides of the same subject (left lower limb), showing (**a**) one, (**b**) two and (**c**) three activation intervals within the gait cycle

EMG signals were high-pass filtered (cut-off frequency of 20 Hz) and then processed by a double-threshold statistical detector [20], embedded in the Step32 system, that provided the onset and offset time instants of muscle activity in a completely user-independent way. This detector was applied to the raw EMG signal and, hence, it did not require any envelope detection (Fig. 3). The detection technique consisted of selecting a first threshold ζ and observing *m* successive samples: if at least *r*_0_ out of successive *m* samples were above the first threshold ζ, the presence of the signal was acknowledged. In this approach, the second threshold was represented by *r*_0_. Thus, the behavior of the double-threshold detector was determined by three parameters: the first threshold ζ, the second threshold r_0_, and the length of the observation window *m*. Their values were selected to jointly minimize the value of false-alarm probability and maximize probability of detection for each specific signal-to-noise ratio. The setting of the first threshold, ζ, was based on the assessment of the background noise level, as a necessary input parameter. Furthermore, the double-threshold detector required to estimate the signal-to-noise ratio in order to fine tune the second threshold, r_0_. The values of the background noise level and the signal-to-noise ratio, necessary to run the double-threshold algorithm, were estimated for each signal by Step32 system, using the statistical approach described in [21]. The length duration of the observation window, *m*, was set equal to 30 ms, that was considered a suitable value for the study of muscle activation in gait analysis [20].

The co-contraction of ankle joint muscles was assessed calculating: 1) the percentage of cycles showing a simultaneous activation of TA and GL, within a specific gait phase (H, F, P and swing), 2) the average co-contraction duration in these cycles (TA/GL simultaneous activation expressed as % GC).

The EMG activation patterns of TA, GL, PL, RF, and LH, bilaterally, were obtained in the two testing conditions of a) walking and b) walking and texting. In previous studies we found that human locomotion is not characterized by a single “preferred” pattern of muscle activation, but rather by up to 4–5 distinct EMG patterns, each distinguished by a different number of activation intervals occurring within a gait cycle [12, 13]. As an example, in Fig. 3, three different activation patterns of GL were displayed, observed in three different strides extracted from the same walk, showing 1, 2 and 3 activations, respectively. With this example, we wanted to clarify that EMG variability must be properly handled, and that it might be incorrect to apply ensemble averages over EMG patterns showing a different number of activation intervals. Hence, the muscle activation timing was averaged across the various strides of a subject’s gait, bundling together only EMG patterns sharing the same number of activation intervals within the gait cycle. EMG patterns sharing the same number of activation intervals were named “activation modalities” [12]. To evaluate the “representativeness” of each activation modality, it was calculated its occurrence frequency, i.e. in how many strides a specific modality was observed with respect to the total number of strides. The muscle activation timing over the population was evaluated separately for each activation modality. The number of subjects showing muscle activity at each specific percent of the gait cycle was gray-level coded, with “black” meaning that all subjects showed muscle activity and “white” meaning that none of the subjects activated the muscle. Matlab® custom routines were used to process the data.

### Statistical analysis

All data distributions were tested for normality with a Kolmogorov–Smirnov test. For each of them, the null hypothesis could not be rejected at a significance level α = 0.05. For each spatio-temporal and kinematic parameter, a paired *t*-test (α = 0.05, 2 tails) was applied to determine if there was a significant difference between the conditions of “walking” and “walking while texting”. To compare EMG timing between conditions we used 1-way MANOVA approach (Wilk’s Lambda statistics): for each muscle, we considered as dependent variables the onset and offset instants of each activation interval, in each modality. Post hoc univariate analysis was performed with t-tests (α = 0.05, 2 tails) when the MANOVA outcome was significant (*p* < 0.05), to explore in which modality and for which specific activation interval there was a difference between conditions.

## Results

All subjects except one typed the message using both hands. The average typing speed was 80 ± 13 characters/minute.

### Spatio-temporal parameters

Texting while walking slowed subjects’ gait speed (Table 1), reducing both their cadence and stride length. Conversely, the double support period and CV of stride time increased. For what concerns the duration of the sub-phases of stance, the flat foot contact increased, and the push-off decreased. Although all the mentioned differences between single-task and dual-task conditions are significant, the absolute effect size is small. In particular, focusing on the variables characterizing gait stability, it can be noticed that the double support period changed only by 2 % GC under dual-task condition, and the CV of stride time by 0.5 %.

**Table 1:** Gait parameters in single-task and dual-task conditions, and dual-task effect

	Walking	Walking and texting	*p*-value	DTE^b^
	(single task)	(dual task)		
Spatio-temporal parameters				
Gait speed (m/s)	1.30 ± 0.12	1.17 ± 0.10	<0.001	10.0 ± 3.8 %
Cadence (strides/min)	54.9 ± 2.9	52.4 ± 3.9	<0.001	4.6 ± 3.1 %
Stride length (m)	1.42 ± 0.14	1.34 ± 0.11	<0.001	5.6 ± 3.5 %
Double support (% GC)	11.2 ± 2.7	13.3 ± 2.3	<0.001	23 % ± 20 %
Stride-to-stride variability				
CV^a^ of stride time (%)	1.86 ± 0.42	2.33 ± 0.63	0.008	28 ± 34 %
Sub-phases of stance (duration)				
H, Heel contact (% GC)	6.6 ± 2.0	6.9 ± 3.3	0.4	-
F, Flat foot contact (% GC)	26.4 ± 4.0	30.0 ± 4.3	<0.001	14 ± 8 %
P, Push off (% GC)	22.6 ± 4.0	19.8 ± 3.4	<0.001	12 ± 6 %

Values are mean ± standard deviation over the population. The left and right side values were averaged

^a^CV: Coefficient of Variation = (standard deviation/mean) × 100

^b^DTE: Dual Task Effect = [|(single-task $-$ dual-task)|/single-task]×100

### Ankle and knee kinematics

**Fig. 4:**
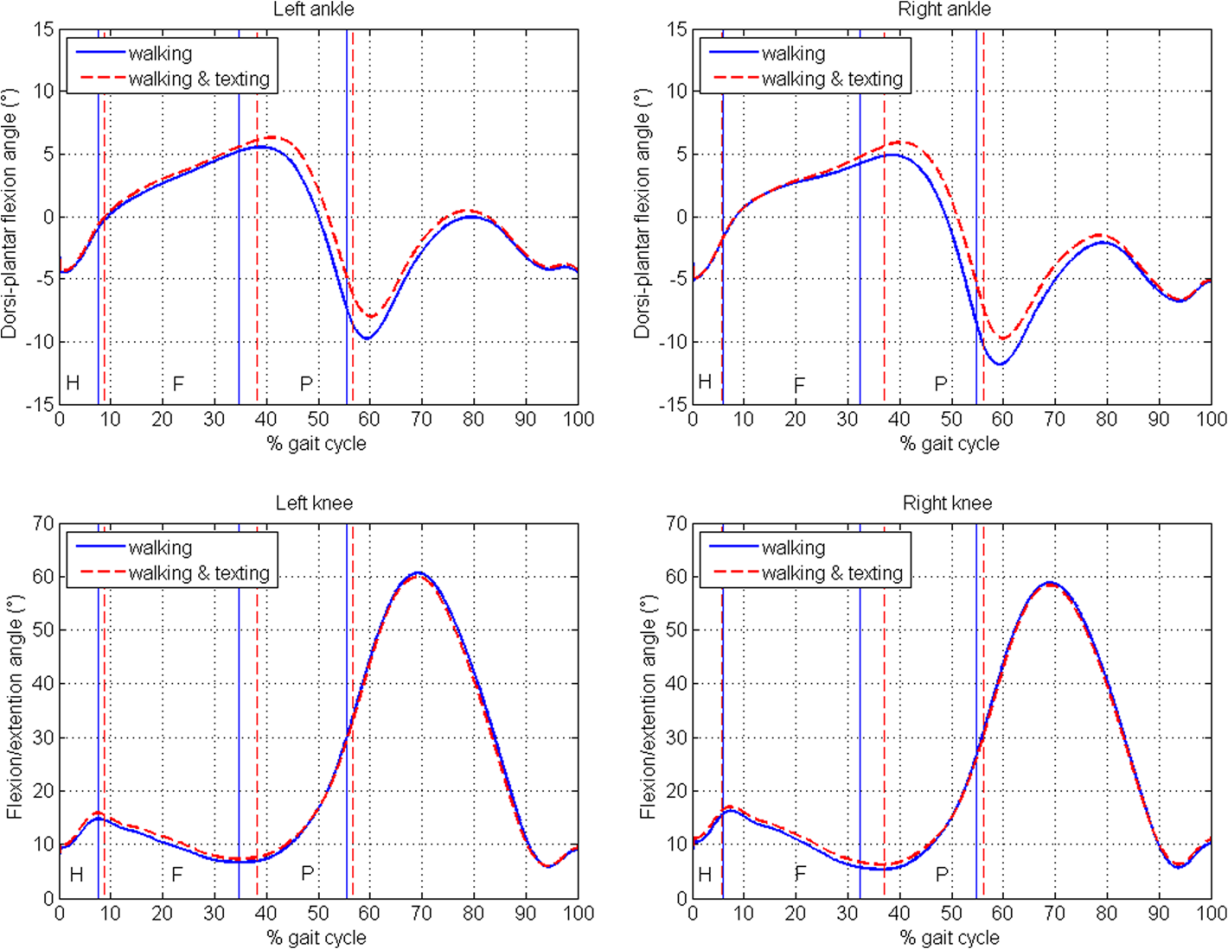
Ankle and knee joint kinematics. Ankle and knee joint kinematics for the left and right side are represented (multiple strides were averaged for each subject and then the global average across subjects was considered). Two conditions are depicted: walking (*blue continuous line*) and walking & texting (*red dashed line*). The sub-phases of stance (H: heel contact, F: flat-foot contact, P: push-off) are delimited by vertical lines for both conditions

The joint kinematics of the two test conditions were very similar (Fig. 4). Visually, they were practically superimposed at initial contact. A slightly increased ankle dorsi-flexion followed by a slightly reduced plantar-flexion in the “walking and texting” condition could be noticed, but differences in kinematic peak values, always smaller than 2 deg, were never statistically significant (see Table 2).

**Table 2:** Kinematic angles

	Walking	Walking and texting	*p*-value
	(single task)	(dual task)	
Ankle			
Max dorsi-plantar flexion (°)	5.4±2.3	6.3±3.1	0.39
Min dorsi-plantar flexion (°)	−11.1±4.8	−9.2±5.1	0.35
Knee			
First peak of knee flexion (°)	15.7±4.9	16.6±4.9	0.66
Max of knee flexion (°)	60.0±5.5	59.4±6.0	0.81

Values are mean ± standard deviation over the population. The left and right side values were averaged

### Muscle activation patterns

**Fig. 5:**
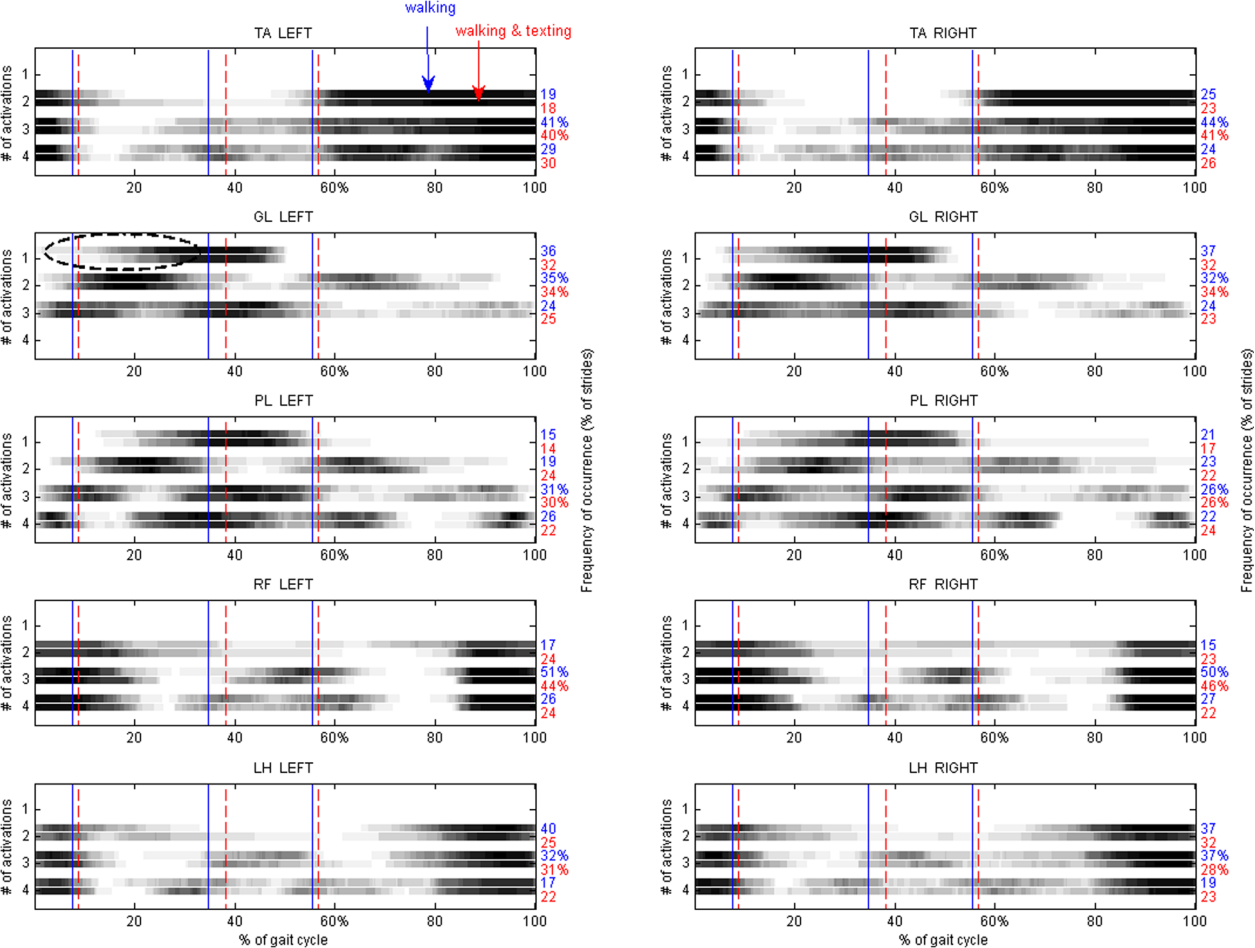
Muscle activation patterns. Muscle activation patterns of tibialis anterior (TA), gastrocnemius lateralis (GL), peroneus longus (PL), rectus femoris (RF) and lateral hamstring (LH), left and right side. Patterns with 1 to 4 activation intervals within the gait cycle are represented (only the patterns occurring in at least 10 % of the gait cycles are depicted). The percentage frequency of occurrence of each pattern is reported on the right-hand side of each plot. For each pattern of activation, the upper bar represents the “walking” single-task, while the lower bar the “walking and texting” double-task. Horizontal bars are grey-level coded in order to portray the number of subjects whose muscle was active at a specific percent of the gait cycle. Black: all the subjects activated the muscle, white: none of the subjects activated the muscle. The gait phases are delimited by vertical lines (blue: walking; red: walking and texting). The only statistically signicant difference between conditions was emphasised with an ellipse

There were no significant differences between single and dual-task conditions, except for the left GL muscle (MANOVA *p* = 0.02). The post hoc analysis showed that, in the 1-activation modality, the muscle activation onset was delayed under dual-task (21 ± 6.4 % GC vs. 16.4 ± 7.6 % GC, *p* < 0.001). A pictorial representation of the muscle activation patterns, obtained separating the different activation modalities, was reported in Fig. 5.

### Ankle muscles co-contraction

**Fig. 6:**
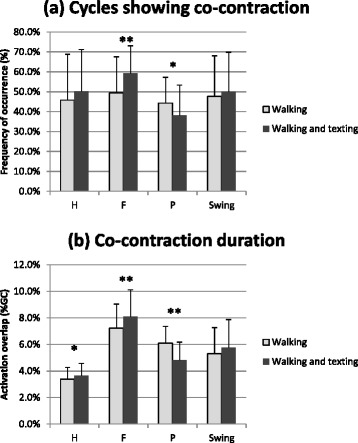
Co-contraction of ankle antagonist muscles. Co-contraction between tibialis anterior (TA) and gastrocnemius lateralis (GL). (**a**) Percentage of cycles with TA/GL co-contraction during heel contact (H), flat foot contact (F), push off (P) and swing. (**b**) Co-contraction duration (expressed as % of gait cycle). Significant differences between walking conditions are indicated as *(*p* < 0.05) or **(*p* < 0.001)

Under dual-task, the co-contraction of TA and GL was augmented in some of the sub-phases of stance, and it was diminished in others. More specifically, during the H-phase, the percentage of cycles showing co-contraction was augmented, although the statistical significance was not reached (Fig. 6). In these cycles, the co-contraction duration was slightly increased (from 3.4 to 3.6 %, *p* = 0.03). During the F-phase, the percentage of cycles showing co-contraction was augmented (from 49.4 to 59.4 %, *p* < 0.001). Also the co-contraction duration was increased (from 7.2 to 8.1 %, *p* < 0.001). During the P-phase, the percentage of cycles showing co-contraction was diminished (from 44.3 to 38.2 %, *p* = 0.04). Also the co-contraction duration was diminished (from 6.1 to 4.8 %, *p* < 0.001). In swing, there were no significant changes in the TA/GL co-contractions.

## Discussion

### Spatio-temporal parameters

The task assigned to participants involved both “thinking” and “typing” while walking, as it happens in the everyday-life use of a smartphone. Walking-typing most probably increased the visuospatial attentional load, while walking-thinking allowed the participant to spend more time looking at the path instead of the display. This might explain the small velocity reduction observed. On the average, young adults slowed their gait speed only by 10 % when texting while walking. In literature, it was reported a reduction of 23 % when typing a phrase appearing on the smartphone screen [6] and a reduction of 32 % when typing a pre-assigned sentence [3]. On the other hand, it was reported a reduction of 17 % when writing an email while walking, answering a question previously posed [22], a protocol more similar to the one we used, in that it implies also “thinking” and not only “typing”. However, differently from [22], we analyzed a prolonged task lasting 3 min along a 15-m walkway, instead of 3 separate 10-m trials. It was not possible to establish if participants were writing at the same typing speed throughout, but we checked that they maintained a stable gait speed among the walkway passages.

The average typing speed that we obtained was much slower (80 ± 13 chars/min.) than in other studies (222 ± 45 chars/min) [6]. This is not surprising since the secondary task (texting) was different. In [6] participants were instructed to type the phrase that appeared on the screen “as quickly and as accurate as possible into the textbox below the phrase”, while our participants were engaged also in a memory effort when asked to describe their activity on the day before the test. Therefore, the slower typing speed may be explained by the fact that we did not chose a pure “typing” task (like typing a predetermined sentence as fast as possible), but a more realistic condition in which the subject also had to think to what he was writing. This slowed the typing speed, but limited to a small amount the gait speed decrease under dual-task (10 %).

Furthermore, our results showed an increase in stride time variability under a dual-task (28 %) higher than that reported (17 %) when analyzing a pure cognitive task (backward counting) [8]. Again this is not surprising since the task that we considered involved not only cognitive resources, but also the integrated use of near and far vision and bimanual coordination.

For what concerned the sub-phases of stance, our results showed that the F-phase was prolonged by 3.6 %GC and that the P-phase was shortened by 2.8 %GC, under dual-task. These small changes may be explained by the gait speed (and stride length) reduction.

### Ankle and knee kinematics

Our results did not reveal any significant alterations of the ankle and knee joint kinematics.

### Muscle activation patterns

The muscle activation patterns did not show statistically significant modifications when texting while walking, with the exception of a slightly delayed onset of the left GL, in the first activation modality.

### Ankle muscle co-contraction

Co-contraction is a strategy used by the CNS to achieve movement accuracy by controlling dynamic joint stability, especially during the learning process of a novel task [23–25]. However, the majority of the studies about the role of co-contraction on human motor control focused their attention on the upper limb [26]. Our results showed that the ankle muscle co-contractions were slightly augmented in the H-phase (roughly corresponding to load response) and in the F-phase (mid-stance), when the foot reached the full contact with the floor initiating the single limb stance. Conversely, the co-contractions decreased during the P-phase (terminal stance).

Our results may be interpreted as an increased need of stabilizing the ankle joint during a “critical” phase of the gait cycle, when the body weight was transferred from one leg to the other. The decrease of co-contractions in terminal stance may indicate that the CNS supplied more “attention” to the contralateral limb on whom the weight load was being transferred. Hence, the motor control strategy seemed different in the different phases of the gait cycle: increasing co-contractions when the body load was sustained by a single limb; decreasing co-contractions when both feet were providing a proprioceptive input. This finding was probably not influenced by the walking speed reduction. In fact, previous research demonstrated no modifications in the ankle muscle co-contraction levels when reducing the walking speed by 10 % [27].

Globally, there weren’t any evident trends in data suggesting that those who typed faster (i.e. those that could be argued to be more attentional loaded with the texting task) had larger gait DTE. In cognitive sciences is being debated the concept of “digital natives” [28] to indicate young individuals that have spent their entire lives surrounded by the tools of the digital age, naturally skilled at multitasking. While the concept is new in the field of gait analysis, our results seem to indicate that, overall, the gait modifications due to texting while walking are minimal in young adults. However, we do not interpret our results to mean that texting while walking is a “safe” dual task activity. Safe ambulation in the real world requires appropriate attentional resources to maintain dynamic stability while monitoring for environmental hazards [4, 29] and the difference between laboratory and real-world settings are well documented [5].

### Study limitations

It is very difficult to identify if the effects of texting while walking are due to changes in gait speed between the conditions, or if they are due to the effects of texting. We had no control conditions in which the walking speed was matched. Hence, we cannot exclude that the findings that we obtained could be explained solely by the change in walking speed. Nevertheless, there are no clear trends indicating that participants who reduced more their walking speed showed a correspondingly higher co-contraction increase.

We measured only the average typing speed, and hence we do not know if the participants were writing at the same typing speed throughout. Furthermore, we had no measure of the time participants spent walking-typing vs. walking-thinking. This could be important since walking-thinking would result in more time looking at the path. This evaluation could also be addressed by taking some measure of eye movements to estimate time spent looking at screen vs. path. Future studies may consider including mobile eye-tracker devices to this purpose.

## Conclusions

Young adults engaged in the double task of texting while walking showed minimal modifications to their walking scheme. They slightly reduced their gait speed to safely cope with the task. Gait adaptations in terms of 1) sub-phases of stance, 2) stride-to-stride variability, 3) ankle and knee joint kinematics, 4) muscle activation patterns, and 5) co-contraction of ankle antagonist muscles were comprehensively documented for the first time. We found an increased co-contraction of the ankle antagonist muscles in the “critical” gait phase spanning from load response to mid-stance, phase that corresponds to the body weight transfer from one leg to the other. This seems a CNS adaptation under dual task, responding to an increased need for ankle stabilization.

The methodology described to study the muscle activation patterns and co-contractions by means of statistical gait analysis may be extended to other dual-task studies.

## Abbreviations

CNS: Central nervous system
CV: Coefficient of variation
DTE: Dual task effect
EMG: Electromyography
F: Flat-foot contact
GC: Gait cycle
GL: Gastrocnemius lateralis
H: Heel contact
LH: Lateral hamstring
P: Push off
PL: Peroneus longus
RF: Rectus femoris
TA: Tibialis anterior
